# Reappraising the clinical usability of consolidation-to-tumor ratio on CT in clinical stage IA lung cancer

**DOI:** 10.1186/s13244-022-01235-2

**Published:** 2022-06-17

**Authors:** Dong Woog Yoon, Chu Hyun Kim, Soohyun Hwang, Yoon-La Choi, Jong Ho Cho, Hong Kwan Kim, Yong Soo Choi, Jhingook Kim, Young Mog Shim, Sumin Shin, Ho Yun Lee

**Affiliations:** 1 Department of Thoracic and Cardiovascular Surgery, Chung-Ang University Hospital, Chung-Ang University College of Medicine, Seoul, Korea; 2 Center for Health Promotion, Samsung Medical Center, Seoul, Korea; 3 Department of Pathology and Translational Genomics, Samsung Medical Center, Sungkyunkwan University School of Medicine, Seoul, Korea; 4 Department of Thoracic and Cardiovascular Surgery, Samsung Medical Center, Sungkyunkwan University School of Medicine, 81 Irwon-Ro, Gangnam-Gu, Seoul, 06351 Korea; 5 Department of Thoracic and Cardiovascular Surgery, School of Medicine, Ewha Womans University, Mok-dong Hospital, Seoul, Korea; 6 Department of Radiology and Center for Imaging Science, Samsung Medical Center, Sungkyunkwan University School of Medicine, 81 Irwon-Ro, Gangnam-Gu, Seoul, 06351 Korea; 7 Department of Health Sciences and Technology, SAIHST, Sungkyunkwan University, Seoul, 06351 Korea

**Keywords:** Lung cancer, Ground-glass opacity, Consolidation-to-tumor ratio, Prognosis

## Abstract

**Objectives:**

Ground-glass opacity (GGO) on computed tomography is associated with prognosis in early-stage non-small cell lung cancer (NSCLC) patients. However, the stratification of the prognostic value of GGO is controversial. We aimed to evaluate clinicopathologic characteristics of early-stage NSCLC based on the consolidation-to-tumor ratio (CTR), conduct multi-pronged analysis, and stratify prognosis accordingly.

**Methods:**

We retrospectively investigated 944 patients with clinical stage IA NSCLC, who underwent curative-intent lung resection between August 2018 and January 2020. The CTR was measured and used to categorize patients into six groups (1, 0%; 2, 0–25%; 3, 25–50%; 4, 50–75%; 5, 75–100%; and 6, 100%).

**Results:**

Pathologic nodal upstaging was found in 1.8% (group 4), 9.0% (group 5), and 17.4% (group 6), respectively. The proportion of patients with a high grade of tumor-infiltrating lymphocytes tended to decrease as the CTR increased. In a subtype analysis of patients with adenocarcinoma, all of the patients with predominant micro-papillary patterns were in the CTR > 50% groups, and most of the patients with predominant solid patterns were in group 6 (47/50, 94%). The multivariate analysis demonstrated that CTR 75–100% (hazard ratio [HR], 3.85; 95% confidence interval [CI], 1.58–9.36) and CTR 100% (HR, 5.58; 95% CI, 2.45–12.72) were independent prognostic factors for DFS, regardless of tumor size.

**Conclusion:**

We demonstrated that the CTR could provide various noninvasive clinicopathological information. A CTR of more than 75% is the factor associated with a poor prognosis and should be considered when making therapeutic plans for patients with early-stage NSCLC.

## Key points

- The consolidation-to-tumor ratio (CTR) correlated with nodal upstaging, predominant patterns, and tumor-infiltrating lymphocytes.
- The CTR > 75% is an independent prognostic factor for DFS.
- The CTR is a useful imaging biomarker for early-stage lung cancer.

## Introduction

Along with advances in screening methods, the detection of non-small cell lung cancer (NSCLC) at a very early stage has increased [1]. Surgical resection is the best treatment option for patients with early-stage NSCLC, and the 5-year survival rate among patients with stage IA NSCLC after curative resection has increased by up to 70% [2]. However, decreased pulmonary function postoperatively and the subsequent impaired quality of life are troublesome. In addition, more patients are exposed to the risk of secondary lung cancer as life expectancy continues to grow [3, 4]. Therefore, the importance of limited resection, including segmentectomy and wedge resection, has been emphasized for preserving lung function and improved quality of life [5]. However, compared with a lobectomy, a limited resection could be associated with worse local control and survival rates [6, 7]. As a result, the preoperative noninvasive prognostic stratification of early-stage NSCLC is still taking on added significance, and there are ongoing debates about finding out appropriate candidates for limited resection [8].

The invasive size on pathologic examination and corresponding solid size, excluding ground-glass opacity (GGO), on computed tomography (CT) were emphasized in T categories of the 8th edition of TNM classification [9]. However, the stratification of the prognostic impact of GGO components, which is a distinct imaging manifestation of lung on CT, is controversial. Some studies have reported that the presence of GGO has prognostic significance [10]. Other studies have reported that the consolidation-to-tumor ratio (CTR), the ratio of the maximum diameter of solid portion to the maximum tumor diameter, may provide further information beyond the TNM stage [11, 12]. Accordingly, a randomized trial on limited resection is currently underway to determine whether the CTR can be considered for the treatment of early-stage NSCLC [13]. Furthermore, as notable benefits have recently been demonstrated by adjuvant use of immunotherapy for resected early-stage lung cancer [14], distinct immunogenomic features of the GGO component are also of interest [15].

Therefore, in this study, we aimed to evaluate clinicopathologic and prognostic values of the CTR, examine the extended value of the CTR particularly in the era of immunotherapy, and ultimately provide additional evidence for the clinical management of early-stage NSCLC patients.

## Materials and methods

### Patients

**Fig. 1:**
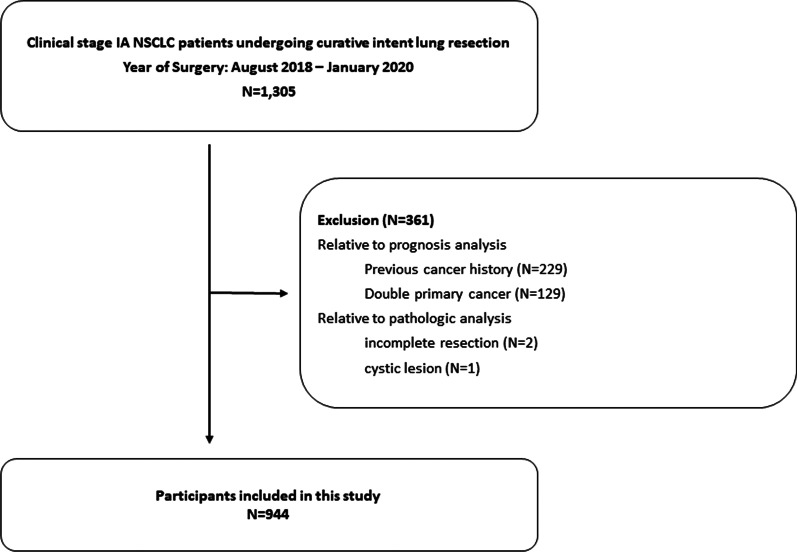
Selection criteria leading to the analytical cohort of patients with clinical stage IA NSCLC

We enrolled 1,305 consecutive patients with clinical stage IA NSCLC who underwent curative-intent lung resection surgery at Samsung Medical Center (Seoul, Korea) between August 2018 and January 2020. We analyzed electronic medical records for patient information up to December 2021. Patients with incompletely resected tumors or fully thin-walled cystic lesions on CT and those with multiple tumors or previous history of cancer were excluded (Fig. 1). Thus, 944 patients were included in the present analysis. All cases were staged according to the 8th edition of the TNM classification for lung cancer [16]. This single-institution retrospective study was approved by our institutional review board with a waiver of informed consent (IRB number 2021–04-167).

### Image acquisition and imaging evaluation

All 944 patients underwent CT imaging before surgery. CT images were obtained with the following parameters: detector collimation, 1.25 or 0.625 mm; 120 kVp; 150–200 mA; and reconstruction interval 1–2.5 mm. All images were displayed at standard mediastinal (window width, 400 Hounsfield Unit [HU]; window level, 20 HU) and lung (window width, 1500 HU; window level, -600 HU) window settings. All CT scans were obtained with 80 cc of contrast material at 2 cc/sec and followed by normal saline 20 cc at 2 cc/sec. Various CT scanners manufactured by different vendors were used, and details of image acquisition were described in a previous study [17].

**Fig. 2:**
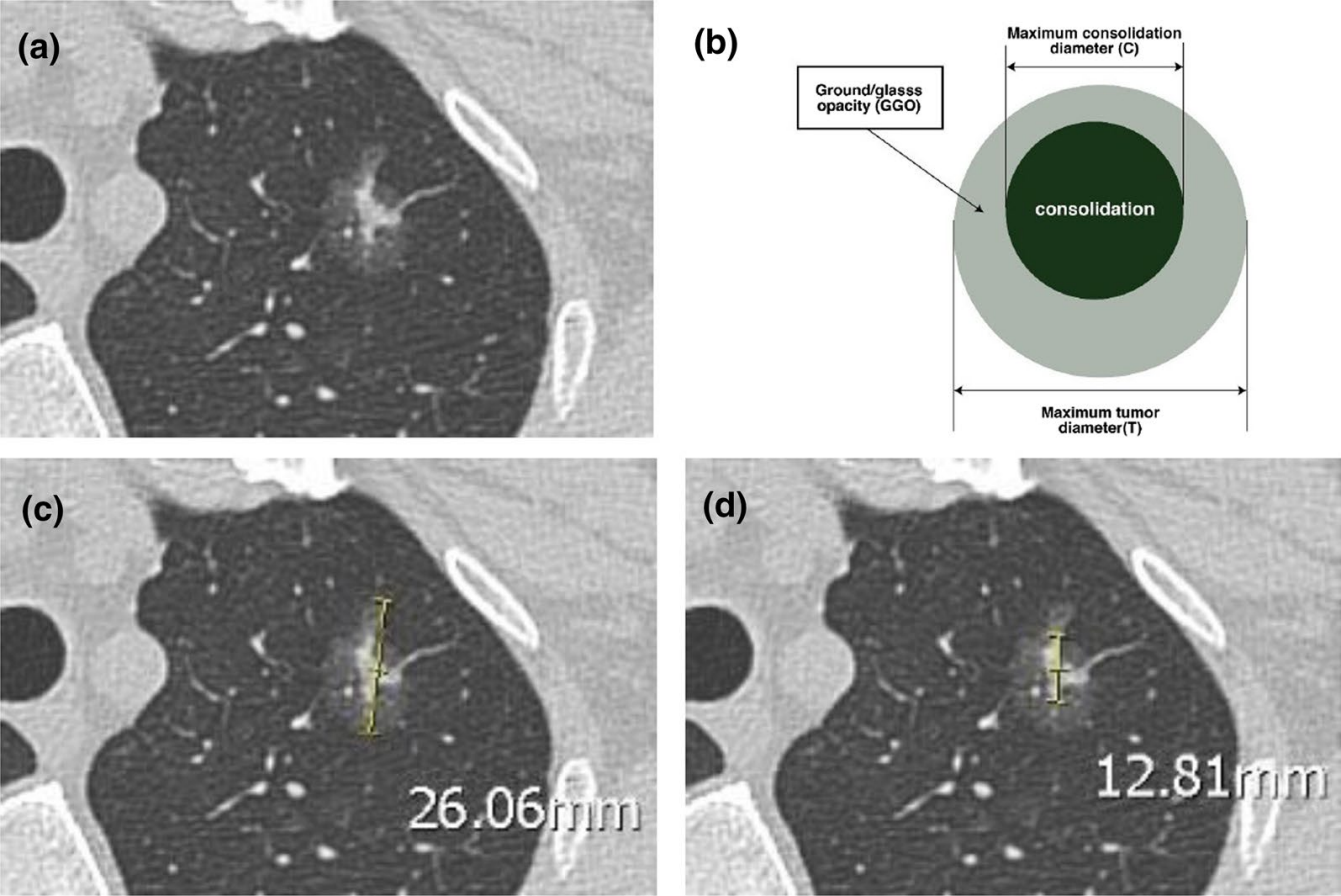
Measurement of the consolidation-to-tumor ratio (CTR). **a** Computed tomography scan shows a part-solid nodule consisting of ground-glass nodule with a solid component. **b** Illustration of measurement of the CTR. **c** The maximum diameter of the entire tumor is 26 mm. **d** The maximum diameter of the solid portion is 12.8 mm

The CTR was measured for each patient. The CTR was defined as the ratio of the maximum diameter of the solid portion divided by the maximum tumor diameter (Fig. 2). The solid portion within the tumor was defined as the area of increased opacification that completely obscured the underlying vascular markings. When measuring the diameter using the lung window setting and multiplanar reconstructions, total and solid diameters were measured in the axial, coronal, and sagittal planes, and the longest total and solid diameters were selected.

Measurement of the CTR in this study was independently performed by two thoracic radiologists (CH Kim and HY Lee). All the clinical information and pathologic results were blinded when reviewing CT scans, and four authors (C.H.K., H.Y.L., D.W.Y., and S.M.S.) reached consensus through discussion in cases of disagreement. Inter-reader agreement was analyzed based on kappa value (0.00–0.20 = poor; 0.20–0.40 = fair; 0.40–0.60 = moderate; 0.60–0.80 = good; and 0.80–1.00 = excellent agreement) [18]. The kappa value of inter-reader agreement was 0.85 for CTR measurement.

### Surgical procedures and pathologic analyses

All of the pulmonary resections were conducted by thoracic surgeons at the Samsung Medical Center. Operative procedures included wedge resection, segmentectomy, and lobectomy as indicated. The surgical extent was selected by considering the size and location of the tumor in each case. If the expected surgical margin was greater than 2 cm, limited resection was considered. A limited resection, with a multidisciplinary approach, was also considered for patients with limited cardiopulmonary function. Systematic mediastinal lymph node dissection that consisted of en bloc resection of all nodes at more than three mediastinal stations and two peribronchial lymph node (LN) stations was conducted in patients undergoing lobectomy or segmentectomy. In the case of wedge resections, lobe-specific selective LN dissection was performed; levels 4 and 7 for right upper and middle lobe; levels 7 and 9 for right lower lobe; levels 5, 6, and 7 for left upper lobe; and levels 7 and 9 for left lower lobe. When LN enlargement was observed or LN metastasis was suspected during the procedure, frozen section biopsies were performed, and systematic LN dissection was undertaken in cases that were positive for malignancy.

Pathologists at the same center examined all intraoperative and postoperative specimens histologically following hematoxylin and eosin staining in reference to the fourth edition of the WHO Classification of Lung Tumors [19]. Total tumor size, invasive size, and histology were evaluated, and histologic subtyping was performed for the primary tumor in a semi-quantitative manner, with each subtype accounting for 5% increments in a total of 100% for each tumor, according to the current IASLC/ATS/ERS lung adenocarcinoma classification system [20]. Grades of tumor-infiltrating lymphocytes (TIL) were assessed using a three-tiered scale designed by an experienced lung cancer pathologist (Y.L. Choi). The percentage of tumor stroma containing mononuclear immune cells, including lymphocyte and plasma cells, was categorized into three grades: low, moderate, and high.

### Clinical follow-up and outcomes

Patients were followed up regularly every 3 months for the first 2 years after surgery, and every 6 months during the next 3 years with an annual CT scan. Depending on the symptoms of the patients, brain CT or brain magnetic resonance imaging and other imaging techniques were used for the detection of recurrence. The primary outcome was disease-free survival (DFS) according to the CTR, and DFS was calculated from the date of surgery to the date of recurrence, death, or last follow-up.

### Statistical analysis

Statistical analyses were performed using *R* studio (version 1.4.1106). Continuous data are presented as means ± standard deviations or medians with interquartile ranges (IQRs), and categorical variables are described as frequencies with percentages. DFS was estimated using the Kaplan–Meier method and compared by the log-rank test across the different groups. Duration of survival was estimated from the time of lung resection surgery to the date of recurrence or death, or the end of the study (December 2021). Univariate and multivariate analyses with Cox proportional hazards models were used to find hazard ratios (HRs) adjusted for clinical and pathologic covariates. A *p* value of less than 0.05 was considered significant.

## Results

### Clinical characteristics

Table 1 presents details of clinical characteristics. Of the 944 patients in this study, the numbers of patients with clinical stage IA1, IA2, and IA3 disease were 280 (29.7%), 350 (37.1%), and 314 (33.3%), respectively. The distribution of patients according to the CTR was: 9.4% (0%), 8.6% (0–25%), 8.8% (25–50%), 12.3% (50–75%), 21.3% (75–100%), and 39.6% (100%). The median follow-up period was 30.8 months (IQR: 26.5–35.5 months), and the number of recurrences or deaths for any reason was 88.

**Table 1:** Patient baseline characteristics

Characteristic	*c*/*t* ratio							*p*-value
Variable	Overall (*n* = 944)	0% (*n* = 89)	0–25% (*n* = 81)	25–50% (*n* = 83)	50–75% (*n* = 116)	75–100% (*n* = 201)	100% (*n* = 374)	
Sex, male	444 (47.0%)	36 (40.5%)	36 (44.4%)	39 (47.0%)	47 (40.5%)	77 (38.3%)	209 (55.9%)	0.001
Age, years	61.9 ± 10.3	59.8 ± 8.5	60.2 ± 8.9	60.6 ± 9.6	61.1 ± 9.6	62.0 ± 10.1	63.2 ± 11.2	0.107
Smoking status								0.002
Never smoker	524 (55.5%)	61 (68.5%)	46 (56.8%)	45 (54.2%)	71 (61.2%)	122 (60.7%)	179 (47.9%)	
Ever smoker	420 (44.5%)	28 (31.5%)	35 (43.2%)	38 (45.8%)	45 (38.8%)	79 (39.3%)	195 (52.1%)	
ECOG (*n* = 933)								
0,1	931 (99.8%)	89 (100.0%)	81 (100.0%)	81 (100.0%)	114 (100.0%)	197 (99.5%)	369 (99.7%)	0.899
2,3	2 (0.2%)	0 (0.0%)	0 (0.0%)	0 (0.0%)	0 (0.0%)	1 (0.5%)	1 (0.3%)	
*Tumor size*								
Total (mm, mean ± SD)	21.7 ± 7.1	19.2 ± 6.1	19.2 ± 6.1	21.6 ± 7.8	24.6 ± 9.1	22.4 ± 7.6	21.5 ± 5.8	0.000
Solid portion (mm, mean ± SD)	15.4 ± 9.2	0	3.2 ± 1.7	8.4 ± 3.6	15.2 ± 5.9	18.8 ± 6.4	21.5 ± 5.8	0.000
*Clinical stage*								
IA1	280 (29.7%)	89 (100%)	81 (100%)	64 (77.1%)	27 (23.3%)	17 (8.5%)	2 (0.5%)	0.000
IA2	350 (37.1%)	0	0	19 (22.9%)	68 (58.6%)	110 (54.7%)	153 (40.9%)	
IA3	314 (33.3%)	0	0	0	21 (18.1%)	74 (36.8%)	219 (58.6%)	
*Surgery type*								
Wedge resection	145 (15.4%)	33 (37.1%)	19 (23.5%)	21 (25.3%)	15 (12.9%)	15 (7.5%)	42 (11.2%)	0.000
Segmentectomy	99 (10.5%)	15 (16.9%)	17 (21.0%)	14 (16.9%)	18 (15.5%)	16 (8.0%)	19 (5.1%)	
Lobectomy	700 (74.2%)	41 (46.1%)	45 (55.6%)	48 (57.8%)	83 (71.6%)	170 (84.6%)	313 (83.7%)	
Follow-up period (months)	30.8 (26.5–35.5)	31.8 (27.1–36.1)	28.8 (23.2–29.7)	32.4 (27.3–36.7)	30.4 (26.8–35.7)	30.8 (26.5–35.9)	30.8 (26–35.4)	0.275

### Pathologic characteristics

**Fig. 3:**
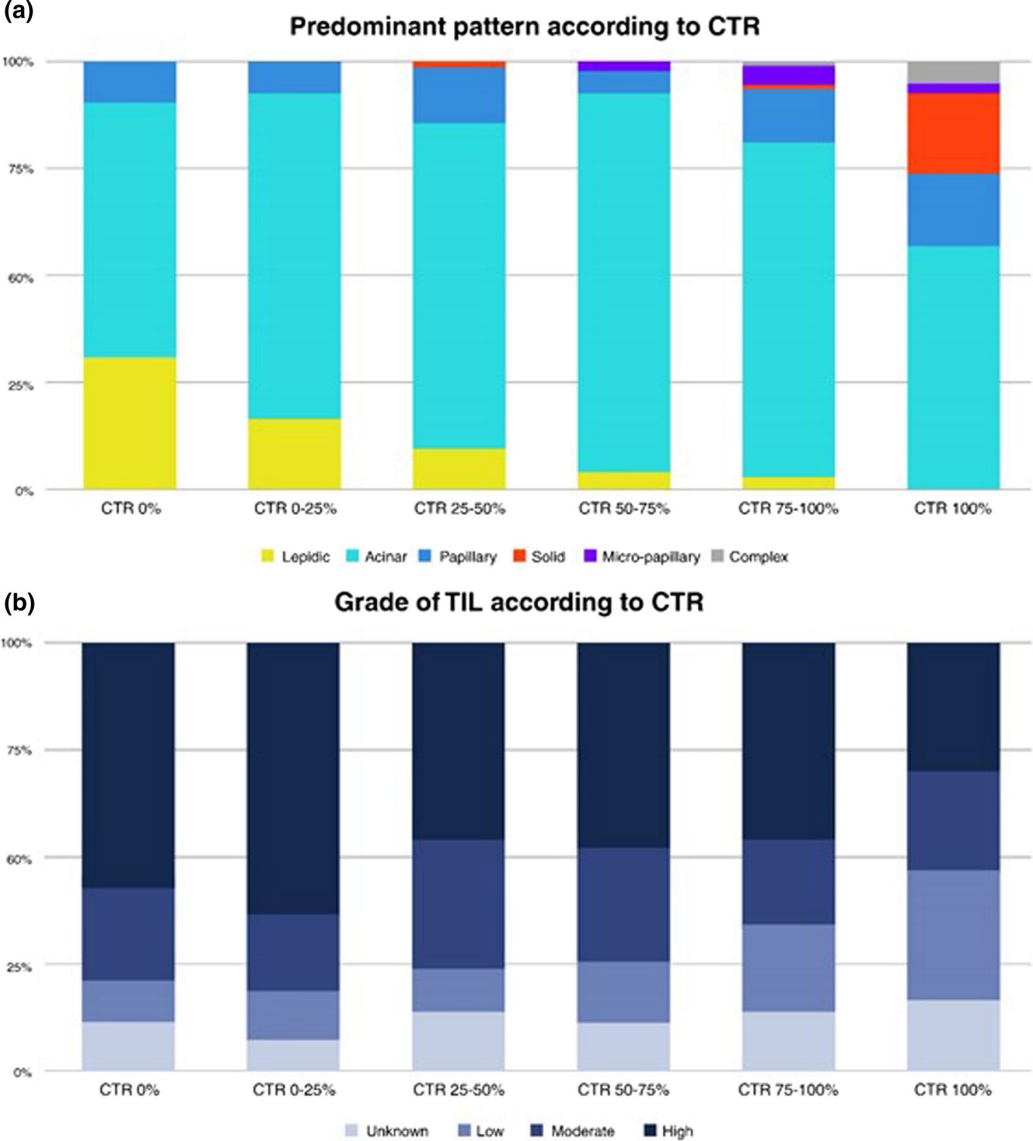
**a** The proportion of predominant patterns according to the CTR in clinical stage IA patients with adenocarcinoma; **b** the proportion of TIL grade according to the CTR in clinical stage IA patients

Table 2 presents details of pathologic characteristics according to quartile CTR. Pathologic invasive sizes increased gradually as the CTR increased. The most common tumor histology was adenocarcinoma (*n* = 852, 90.7%), followed by squamous cell carcinoma (*n* = 66, 7%), and CTR of all squamous cell carcinomas were above 75% [CTR 75–100% group, 5/66 (7.6%); CTR 100% group, 61/66 (92.4%)]. Among adenocarcinoma, the acinar pattern was the predominant histologic pattern in all groups and showed a wide range (56.5%-87.7%). The proportion of patients with lepidic predominant pattern decreased gradually as the CTR increased, from 31% of the CTR 0% group to 0.8% of the CTR 100% group. All of the patients with micro-papillary predominant patterns were in the CTR > 50% groups and ranged from 1.9–4.4%. Patients with solid predominant patterns were in the CTR > 25% groups, and most of them were in the CTR 100% group (47/50, 94%) (Fig. 3a).

**Table 2:** Pathological characteristics of patients

Variable	Consolidation-to-tumor ratio							
	Overall (*n* = 944)	0% (*n* = 89)	0–25% (*n* = 81)	25–50% (*n* = 83)	50–75% (*n* = 116)	75–100% (*n* = 201)	100% (*n* = 374)	
*Pathologic size*								
Total Size (mm, mean ± SD)	20.8 ± 7.8	17.1 ± 5.9	17.6 ± 6.8	19.2 ± 7.3	22.5 ± 9.1	22.2 ± 8.1	21.5 ± 7.3	0.036
Invasive size (mm, mean ± SD)	18.9 ± 8.3	12.2 ± 7.0	13.2 ± 6.6	14.8 ± 6.6	19.3 ± 9.0	21.0 ± 7.9	21.4 ± 7.4	0.014
*Histologic type*								
ADC	852 (90.3%)	89 (100.0%)	81 (100.0%)	83 (100.0%)	114 (98.3%)	194 (96.5%)	291 (77.8%)	0.000
SqCC	66 (7.0%)	0 (0.0%)	0 (0.0%)	0 (0.0%)	0 (0.0%)	5 (2.5%)	61 (16.3%)	
Others	26 (2.8%)	0 (0.0%)	0 (0.0%)	0 (0.0%)	2 (1.7%)	2 (1.0%)	22 (5.9%)	
*Predominant histologic pattern of ADC (n = 787)*								0.000
Lepidic	60 (6.4%)	26 (31.0%)	13 (16.9%)	8 (10.5%)	5 (4.7%)	6 (3.3%)	2 (0.8%)	
Acinar	548 (58.1%)	50 (59.5%)	58 (75.3%)	57 (75.0%)	93 (87.7%)	142 (78.0%)	148 (56.5%)	
Papillary	97 (10.3%)	8 (9.5%)	6 (7.8%)	10 (13.2%)	6 (5.7%)	22 (12.1%)	45 (17.2%)	
Solid	50 (5.3%)	0 (0.0%)	0 (0.0%)	1 (1.3%)	0 (0.0%)	2 (1.1%)	47 (17.9%)	
Micro-papillary	16 (1.7%)	0 (0.0%)	0 (0.0%)	0 (0.0%)	2 (1.9%)	8 (4.4%)	6 (2.3%)	
Complex (cribriform etc.)	16 (1.7%)	0 (0.0%)	0 (0.0%)	0 (0.0%)	0 (0.0%)	2 (1.1%)	14 (5.3%)	
Grade of predominant histologic pattern of ADC* (n = 787)								0.000
Low (Lepidic)	60 (7.6%)	26 (31.0%)	13 (16.9%))	8 (10.5%)	5 (4.7%)	6 (3.3%)	2 (0.8%)	
Intermediate (Acinar, Papillary)	645 (82.0%)	58 (69.0%)	64 (83.1%)	67 (88.2%)	99 (93.4%)	164 (90.1%)	193 (73.7%)	
High (Solid, Micro-papillary, Complex)	82 (10.4%)	0 (0%)	0 (0%)	1 (1.3%)	2 (1.9%)	12 (6.6%)	67 (25.6%)	
*Pathologic* *N stage*								0.000
*N*0	837 (88.7%)	84 (94.4%)	77 (95.1%)	81 (97.6%)	113 (97.4%)	181 (90.1%)	301 (80.5%)	
*N*1	44 (4.7%)	0 (0.0%)	0 (0.0%)	0 (0.0%)	1 (0.9%)	11 (5.5%)	32 (8.6%)	
*N*2	41 (4.3%)	0 (0.0%)	0 (0.0%)	0 (0.0%)	1 (0.9%)	7 (3.5%)	33 (8.8%)	
Unknown	22 (2.3%)	5 (5.6%)	4 (4.9%)	2 (2.4%)	1 (0.9%)	2 (1.0%)	8 (2.1%)	
*Tumor-infiltrating lymphocyte (TIL)*								
Unknown	137	11 (12.4%)	6 (7.4%)	12 (14.5%)	14 (12.1%)	30 (14.9%)	64 (17.1%)	
Low	189	8 (9.0%)	9 (11.1%)	8 (9.6%)	16 (13.8%)	39 (19.4%)	109 (29.1%)	
Moderate	218	19 (21.3%)	15 (18.5%)	25 (30.1%)	31 (26.7%)	41 (20.4%)	87 (23.3%)	
High	400	51 (57.3%)	51 (63.0%)	38 (45.8%)	55 (47.4%)	91 (45.3%)	114 (30.5%)

Pathologic nodal upstaging was found in 9% of the study population, and the CTR of all of them was above 50%. The percentage of total nodal upstaging were 1.8% (CTR 50–75% group), 9.0% (CTR 75–100% group), and 17.4% (CTR 100% group), respectively. The percentage of *N*2 nodal upstaging were 0.9% (CTR 50–75% group), 3.5% (CTR 75–100% group), and 8.8% (CTR 100% group), respectively.

In the TIL grade analysis, the proportion of patients with high-grade TIL tended to decrease and those with low-grade TIL tended to increase, as the CTR increased (Fig. 3b).

### Disease-free survival analysis

**Fig. 4:**
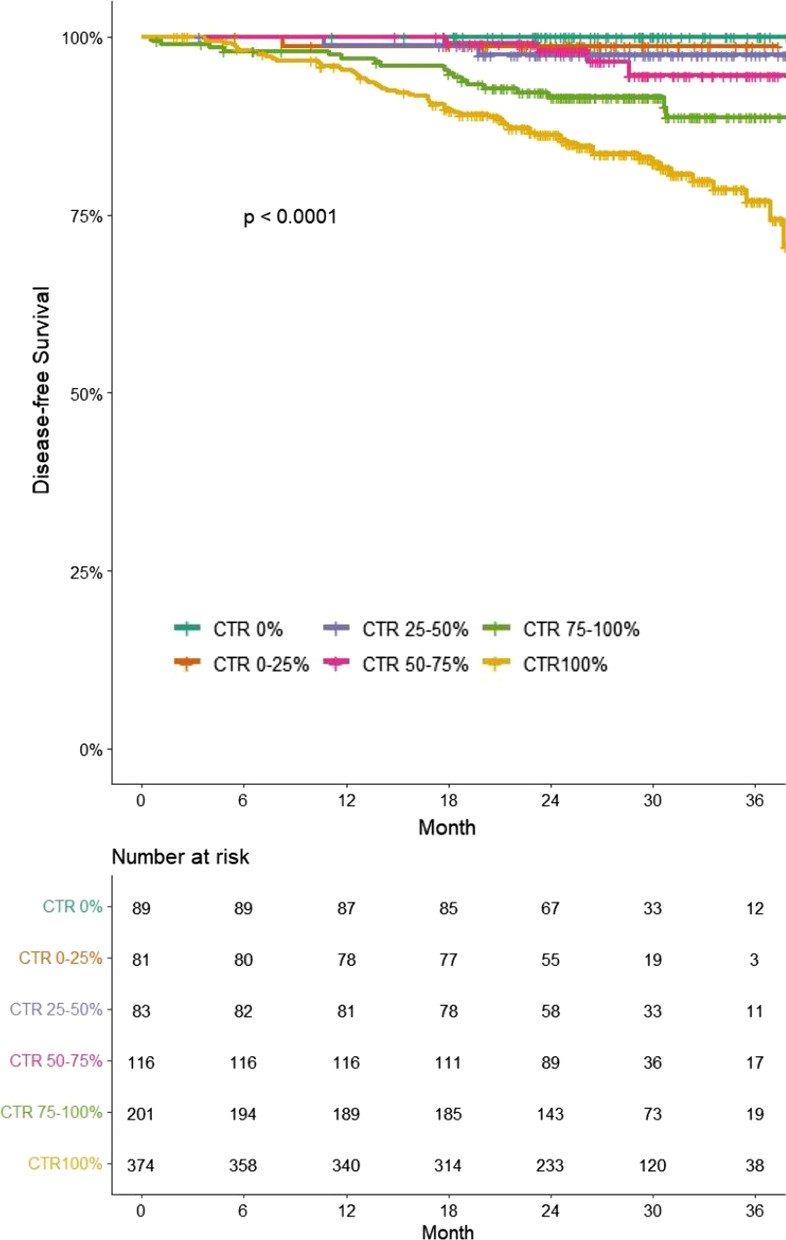
Kaplan–Meier survival curve for disease-free survival according to the CTR divided into six groups of patients in clinical stage IA

**Fig. 5:**
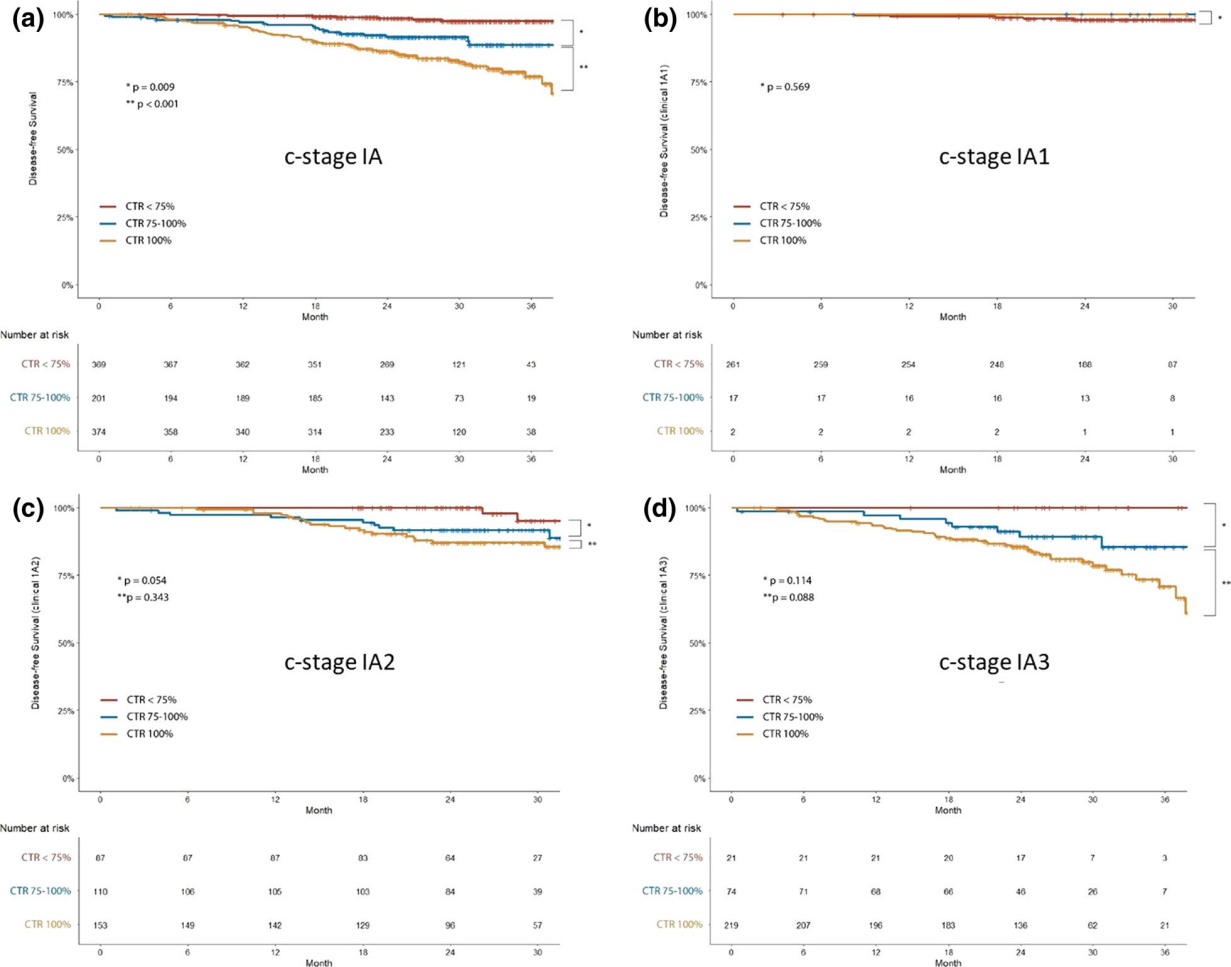
Kaplan–Meier survival curve for disease-free survival according to the CTR divided into three groups of patients in clinical stage IA (**a**), IA1 (**b**), IA2 (**c**), and IA3 (**d**)

The DFS at 30 months according to quartile CTR was as follows: 100% (CTR 0%), 98% (CTR 0–25%), 97.5% (CTR 25–50%), 94.6% (CTR 50–75%), 91.6% (CTR 75–100%), and 83% (CTR 100%) (Fig. 4). Because CTR < 75% had similar survival rates, we divided the patients into three groups: CTR < 75%; CTR 75–100%; and CTR 100%. There were significant differences in DFS between these three groups (Fig. 5a). Figure 5b–d shows DFS graphs of these three groups in stage IA1, IA2, and IA3 patients, respectively.

In the univariate analysis of DFS, male, age, pathologic nodal upstaging, predominant pattern, and CTR were associated with a shorter DFS. The multivariate analysis demonstrated that CTR 75–100% (HR, 3.85; 95% confidence interval [CI], 1.58–9.36) and CTR 100% (HR, 5.58; 95% CI, 2.45–12.72) were independent prognostic factors for DFS (Table 3). In multivariate analysis conducted on patients with adenocarcinoma, CTR 75–100% (HR, 5.34; 95% CI, 1.92–14.80) and CTR 100% (HR, 7.14; 95% CI, 2.67–19.12) were still independent prognostic factors for DFS (Table 4).

**Table 3:** Multivariate Cox regression analysis of disease-free survival (all patients)

Variable	Crude HR (95% CI)	Adjusted HR (95% CI)
Sex (male)	2.18 (1.41–3.37)	1.70 (1.08–2.66)
Age	1.04 (1.02–1.06)	1.03 (1.01–1.05)
*Surgery type*		
Limited resection*	1 (Ref.)	1 (Ref.)
Lobectomy	1.09 (0.66–1.80)	0.50 (0.29–0.87)
Pathologic invasive size	1.78 (1.43–2.22)	1.40 (1.07–1.84)
Histologic type (ADC)	0.43 (0.25–0.72)	1.03 (0.59–1.82)
Pathologic nodal upstaging	4.83 (3.05–7.63)	2.97 (1.81–4.89)
*CTR*		
CTR < 75%	1 (Ref.)	1 (Ref.)
75% < CTR < 100%	4.92 (2.05–11.78)	4.35 (1.78–10.65)
CTR = 100%	9.76 (4.47–21.31)	6.41 (2.82–14.60)

Adjusted for sex, age, surgery type, clinical T stage, histologic type, pathologic nodal upstaging for all subjects

*HR* hazard ratio; *CI* confidence interval; *ADC* adenocarcinoma; and *CTR* consolidation-to-tumor ratio

^*^Limited resection = wedge resection + segmentectomy

**Table 4:** Multivariate Cox regression analysis of disease-free survival (patients with adenocarcinoma)

Variable	Crude HR (95% CI)	Adjusted HR (95% CI)
Sex (male)	2.07 (1.29–3.33)	1.94 (1.17–3.21)
Age	1.03 (1.00–1.06)	1.01 (1.01–1.06)
*Surgery type*		
Limited resection*	1 (Ref.)	1 (Ref.)
Lobectomy	1.59 (0.85–2.96)	0.67 (0.33–1.36)
Pathologic invasive size	1.89 (1.49–2.42)	1.31 (0.96–1.78)
Pathologic nodal upstaging	6.84 (4.20–11.14)	3.69 (2.12–6.40)
Predominant histologic pattern	1.99 (1.17–3.38)	0.79 (0.44–1.44)
*CTR*		
CTR < 75%	1 (Ref.)	1 (Ref.)
75% < CTR < 100%	5.59 (2.20–14.17)	5.36 (1.95–14.77)
CTR = 100%	11.01 (4.71–25.72)	6.93 (2.65–18.17)

Adjusted for sex, age, surgery type, clinical T stage, histologic type, pathologic nodal upstaging, predominant histologic pattern for all subjects

*HR* hazard ratio; *CI* confidence interval; *ADC* adenocarcinoma; and *CTR*: consolidation-to-tumor ratio

^*^Limited resection = wedge resection + segmentectomy

## Discussion

Tumor size is a strong predictor for the prognosis of lung cancer, and this significance was already emphasized by the discrimination of 1 cm interval in the revision of the TNM classification system from the 7th to 8th edition [21]. The 8th edition of the TNM classification recommended using the invasive tumor size as a T-descriptor, excluding the lepidic pattern, which is regarded as a noninvasive growth pattern. Accordingly, GGO, which generally corresponds to lepidic architecture, was excluded in clinical staging [9]. In addition, it has been recently reported that the presence of a GGO component could represent an independent predictor of good prognosis, regardless of the invasive features of the lesion [10, 22]. A GGO is a unique manifestation of lung cancer, which is attributed to the exceptional environment of lung mostly comprised of air [23]. A concept of the CTR, which reflects the ratio of solid portion and GGO, has arisen from this unique characteristic of lung, and the clinical significance of this concept was supported by several studies [24, 25]. In one step further, we attempted to explore the relationship of the CTR with various pathological criteria known to be associated with the prognosis, along with DFS. In addition, we tried to investigate the potential value of the CTR in the era of immunotherapy for NSCLC.

From our findings, the invasive tumor size and prevalence of LN metastasis significantly increased with increasing CTR. In addition, higher CTR groups were more likely to have high-grade histologic patterns. The patient with CTR less than 0.75 had excellent survival with a 30-month DFS of approximately 97.4% compared to 86.1% of those with a CTR of more than 0.75, and the CTR was independent of the prognostic factor for DFS after adjusting for age, sex, pathologic invasive size, histologic type, and pathologic nodal upstaging.

Okubo et al. suggested that GGO features identified by the CT can sometimes be seen in tumors with a non-lepidic pattern [26]. Similarly, in our study, the predominant pattern in the CTR 0% group comprised 58.1% of the acinar pattern, and the proportion of patients with lepidic predominant pattern decreased as the CTR increased. With regard to the high-grade pattern, the percentage of this pattern increased as the CTR increased and was highest in the CTR 100% group. However, the micro-papillary pattern and solid pattern showed a different distribution. The micro-papillary pattern was only observed in the groups with a CTR of more than 50%, and the proportion of this pattern was highest in the group with a CTR of 75–100%. On the other hand, complex and solid patterns were extremely rare in the CTR < 100% groups. Considering that these two patterns had different cancer evolutional properties and prognostic values [27, 28], an attempt to distinguish the proportion of these two patterns based on CTR may have clinical implications, and a further confirmative study with a large cohort would be necessary.

In this study, pathologic nodal upstaging was observed only in tumors with CTR > 50%, and 17.4% of patients with CTR of 100% had mediastinal nodal metastasis. These results were in line with a previous study that CTR of more than 75% was a significant predictor of mediastinal nodal metastasis in patients with NSCLC of a total size of 3 cm or less [29]. Although the ACOSOG Z0030 trial showed that LN dissection did not improve survival compared to LN sampling in early-stage NSCLC [30], systematic complete LN dissection is required for the patients who have a high probability of occult LN metastasis. We believe that CTR could be a potential factor in planning surgical procedures involving LN dissection.

One interesting finding in the current study was that the proportion of patients with a high level of TIL decreased as the CTR increased. These findings were consistent with previous studies by Rosenthal et al. [31] and Nelson et al. [32]. Rosenthal et al. suggested that the immune-microenvironment exerts a strong selection pressure in early-stage NSCLC, producing multiple routes to immune evasion. A study by Rosenthal et al. showed that the level of immune infiltration decreased as immune evasion increased. Nelson et al. reported that the density of the cytotoxic T cell and natural killer cells was lower within radiographically solid lesions compared to the GGO lesion. Our results showed that the CTR could represent tumor evolution from pre-invasive or less invasive lung cancer to invasive lung cancer, and subsequent immune escape. In the current situation, where there are attempts to use immunotherapy for resectable lung cancer [14, 33], there is a need for further confirmative studies to find the relationship between CTR and TIL and establish the value of the CTR.

Our study has several limitations. First, it was a retrospective study performed at a single institution and follow-up periods were insufficient. Second, inter-observer variability, including variance in measurement of the CTR, can be a limitation in the application of our study to clinical practices. Third, heterogeneity of imaging protocols, which can arise from use of different scanners with differences in slice thickness, can affect the accuracy of CTR measurements. The thinnest slice thickness (1 mm) could provide the most consistent results [34]. However, referring to the CT techniques used in both the National Lung Screening Trial [35] and the NELSON trials [36], our study included images with a slice thickness of 2.5 mm or less to maintain adequate imaging quality. Fourth, specific immunohistochemistry analyses were not conducted in tissue samples. However, it is reported that immune-high tumor regions contained greater pathologic estimates of TIL than immune-low regions [31]. Furthermore, assessing TIL using hematoxylin and eosin staining would have value as it is easily integrated into the routine workflow of pathologists.

In conclusion, our study showed radio-pathologic correlation of the CTR and histopathology in stage IA NSCLC. The CTR reflected nodal upstaging, predominant patterns, and TIL, and CTR > 75% was an independent prognostic factor for DFS. The CTR, a useful imaging biomarker, should be considered in management planning in early-stage lung cancer.

## Data Availability

The used data sets analyzed during the study are available from the co-corresponding authors upon request.

## Abbreviations

CT: Computed tomography
CTR: Consolidation-to-tumor ratio
DFS: Disease-free survival
GGO: Ground-glass opacity
HR: Hazard ratio
IQR: Interquartile ranges
LN: Lymph node
NSCLC: Non-small cell lung cancer
TIL: Tumor-infiltrating lymphocytes
